# Boosting Zn^2+^ Storage Kinetics by K-Doping of Sodium Vanadate for Zinc-Ion Batteries

**DOI:** 10.3390/ma17194703

**Published:** 2024-09-25

**Authors:** Mengting Jia, Chen Jin, Jiamin Yu, Shaohui Li

**Affiliations:** School of Materials Science and Engineering, Zhengzhou University, Zhengzhou 450001, China; mengtingjia@stu.zzu.edu.cn (M.J.);

**Keywords:** zinc-ion batteries, vanadium oxides, interlayer doping

## Abstract

Na_5_V_12_O_32_ is an attractive cathode candidate for aqueous zinc-ion batteries (AZIBs) by virtue of its low-cost and high specific capacity (>300 mAh g^−1^). However, its intrinsically inferior electronic conductivity and structural instability result in an unfavorable rate performance and cyclability. Herein, K-doped Na_5_V_12_O_32_ (KNVO) was developed to promote its ionic/electronic migration, and thus enhance the Zn^2+^ storage capability. The as-produced KNVO displays a superior capacity of 353.5 mAh g^−1^ at 0.1 A g^−1^ and an excellent retentive capacity of 231.8 mAh g^−1^ after 1000 cycles at 5 A g^−1^. Even under a high mass of 5.3 mg cm^−2^, the KNVO cathode can still maintain a capacity of 220.5 mAh g^−1^ at 0.1 A g^−1^ and outstanding cyclability without apparent capacity decay after 2000 cycles. In addition, the Zn^2+^ storage kinetics of the KNVO cathode is investigated through multiple analyses.

## 1. Introduction

With the rapidly growing demand in renewable and sustainable energy sources including solar, wind, and tidal energy, aqueous rechargeable batteries have captured considerable interest in the scientific and technical community owing to their cost effectiveness, high ionic conductivity, and intrinsic non-flammability of aqueous electrolytes, having the potential to solve the time and space discontinuity of renewable energy resources [1,2,3,4,5,6,7,8]. Among them, aqueous zinc-ion batteries (AZIBs) are thought to be the most powerful candidates because of the advantages of zinc metal, including high safety, cost-effectiveness, excellent electrochemical stability, high theoretical capacity (820 mAh g^−1^, 5855 mAh cm^−3^), and eco-friendliness [9,10,11,12,13,14,15,16]. Nevertheless, the commercial implementation of AZIBs is limited by the unabilability of suitable cathode materials with both high capacity and durability, because of the high polarization of divalent Zn^2+^ [15,17,18,19,20,21].

To date, many cathodes have been developed to overcome this limitation, including manganese-based materials [7,11,12], vanadium-based compounds [3,22,23], Prussian blue analogs [12,15], and organic compounds [9,12,20,24]. Among them, vanadium-based compounds have attracted positive attention owing to their multivalence states, variable crystalline structures, excellent Zn^2+^ storage performance (generally >300 mAh g^−1^), low cost, and safety [2,25,26]. In comparison with other types of vanadium-based composite cathodes, vanadium oxide cathodes delivered larger reversible specific capacity, better rate-ability, and cycle stability owing to their layered or tunnel structures, which are advantageous for reversible Zn^2+^ insertion–extraction [23,27,28]. In particular, the layered metal vanadates (A_x_V_m_O_n_, A = metal cations) with open framework crystal structures are thought to be a favorable cathode for AZIBs because of the high valence state of vanadium and the enlarged interlayer spacing by the metal cations [17,23,29,30]. For example, Nazar’s group has reported layered 1D Zn_0.25_V_2_O_5_·nH_2_O nanobelts as Zn^2+^ host cathode materials in 2016 [1]. Owing to stabilization by Zn^2+^ and crystalline water, the Zn//Zn_0.25_V_2_O_5_·nH_2_O battery can display an excellent reversible capacity of 300 mAh g^−1^ at 50 mA g^−1^ with an ideal cyclability of 80% retention after 1000 cycles. Mai and colleagues presented a Na_2_V_6_O_16_·1.63H_2_O nanowire cathode [28], which exhibited a superior capacity of 352 mAh g^−1^ with 90% capacity maintenance after 6000 cycles. Cao and co-workers synthesized a polyvanadate-type cathode [29], Na_6_V_10_O_28_, which delivered a reversible capacity of 279.5 mAh g^−1^ after activation and remarkable cyclability. Similarly, a wide variety of vanadium oxides were investigated for AZIBs in recent years, including Ni_0.22_V_2_O_5_·nH_2_O [31], (NH_4_)_2_V_6_O_16_·1.5H_2_O [32], Mn_0.26_V_2_O_5_·nH_2_O [33], NH_4_V_4_O_10_ [17], and Zn_3_V_2_O_7_(OH)_2_·2H_2_O [34]. Great progress has been made in Zn^2+^ diffusion channels, crystal structure design, and Zn^2+^ storage kinetics. However, due to the inferior electronic conductivity and structural collapse caused by the significant volume variation upon Zn^2+^ insertion–extraction cycles, this leads to poor rate capability and unsatisfactory cycling performance.

To tackle these problems, some strategies have been adopted, such as interlayer doping, adding conductive carbon, and vacancy defect engineering [3,35,36]. Among these strategies, interlayer doping is a valuable approach to regulate interlayer spacing, promote ionic–electronic migration, and improve structural stability [17,18,28]. Previous research has proved that both non-metallic ions (NH_4_^+^, H^+^) and metallic ions (Na^+^, K^+^, Zn^2+^, Ca^2+^, Mn^2+^) can be doped into the interlayer or channel of vanadium oxides to improve their diffusion kinetics and electrochemical mechanism [13,17,30,33,34,37]. For instance, Li’s group reported a Mn^2+^-doped layered vanadium oxide AZIB cathode [38], which benefited from the synergistic effects of layered nanostructure and Mn^2+^ doping and greatly enhanced ions–electrons delivery and structural stability, resulting in a superior Zn^2+^ storage performance of 367 mAh g^−1^ and remarkable cyclability after 8000 cycles. Recently, layered and structured Na_5_V_12_O_32_ has been considered to be a promising cathode candidate for AZIBs due to the large interlayer spacing and mixed valence state of V^4+^ and V^5+^, which can improve the ion–electron diffusion kinetics in the AZIB system [27,30]. Unfortunately, similarly to other vanadium oxide cathode materials, the Na_5_V_12_O_32_ suffers from inferior electrical conductivity and repeated lattice expansion and contraction during Zn^2+^ intercalation–deintercalation, resulting in unsatisfactory capacity and cyclability. Therefore, a breakthrough in the effective utilization of layered Na_5_V_12_O_32_ as a high-performance AZIB cathode is urgently needed.

Herein, we reported a K-doped, layered Na_5_V_12_O_32_ (KNVO) nanobelts cathode by the one-pot hydrothermal method. The interlayer doping of K can increase the interlayer spacing, thus boosting the ions–electrons transport kinetics and structural stability of Na_5_V_12_O_32_ (NVO) nanobelts. When applied as a cathode in AZIBs, the as-obtained KNVO electrode exhibits a superior capacity of 353.5 mAh g^−1^ at 0.1 A g^−1^, an outstanding rate capability (185.8 mAh g^−1^ at 10 A g^−1^), and excellent cyclability (no obvious capacity deterioration after 1000 cycles).

## 2. Materials and Methods

### 2.1. Material Synthesis

The synthesis of the KNVO nanobelts was performed as follows: 10 mM of commercial vanadium (V) oxide (V_2_O_5_) powder was added to 60 mL of a 0.5 M oxalic acid solution and then stirred at 25 °C until a transparent blue solution was formed. Then, 856 mg sodium nitrate (NaNO_3_) and 54 mg potassium nitrate (KNO_3_) were added, with stirring for another 1.5 h. The mixture was then poured into a 100 mL Teflon-lined autoclave and hydrothermally treated at 200 °C for 12 h. After natural cooling, the resulting materials were harvested by vacuum filtration and washed several times with deionized water and alcohol, respectively. Finally, the KNVO nanobelts were obtained after drying the filter residue at 100 °C for 10 h.

For the synthesis of the NVO nanobelts, the preparation procedure is same as the KNVO except that the nitrate was changed to 901 mg NaNO_3_. Besides the samples above, the KNVO-2.5 and KNVO-7.5 cathodes were preapred by changing the mass of the KNO_3_ and NaNO_3_ to 27–878 and 81–833 mg, respectively.

### 2.2. Materials Characterization

The crystalline structure and composition were determined by powder X-ray diffraction (XRD, Rigaku Smartlab SE, Tokyo, Japan). Raman spectrum was characterized on a Jobin-Yvon HR800 Raman spectroscope. A scanning electron microscopy (SEM, ZEISS Gemini 300, Jena, Germany) and transmission electron microscopy (TEM, JEOL JEM-F200, Tokyo, Japan) were used to observe the morphologies and nanostructures. The valence state and chemical composition evolution were adopted on an X-ray photoelectron spectroscopy (XPS, Thermo Scientific K-Alpha, Waltham, MA, USA). The Fourier transform infrared spectrum (FTIR) was collected on a FTIR spectrometer (Shimadzu, Prestige 21, Kyoto, Japan).

### 2.3. Electrochemical Measurements

All the batteries were manufactured in coin cells (CR2032 type) in ambient air, and the electrochemical testing was performed on a NEWARE battery testing system at room temperature. The cathodes were created by dispering the active materials, super P carbon black, and polyvinylidene fluoride (PVDF) in a proper amount of *N*-methyl-2-pyrrolidone (NMP) with a mass ratio of 7:2:1 to obtain a homogeneous slurry. The slurry was casted on carbon paper and vacuum dried at 80 °C for 15 h. The Zn//KNVO coin-type cell was constructed using Zn foil, a glass filter (Whatman GF-D), and 3 M of a zinc trifluoromethanesulfonate aqueous solution as the anode, separator, and electrolyte, respectively. The electrolyte added for each coin-type cell was about 80 μL. The Al plastic pouch cell consisted of a 3 × 3.5 cm^2^ Zn foil as the anode, a 3.5 × 4 cm^2^ glass fiber as the separator, and a KNVO-coated stainless-steel film as the cathode. The amount of electrolyte used in the pouch cell was 1.5 mL. The area mass loading of each electrode is about 1.3–1.5 mg cm^−2^. The cyclic voltammograms (CV) profiles and electrochemical impedance spectroscopy (EIS) were tested on a Biologic SP150 electrochemical workstation with CR2032 type coin cells. The galvanostatic intermittent titration technique (GITT) was carried out with 900 s discharge at 0.1 A g^−1^ and 1200 s relax.

## 3. Results and Discussion

**Figure 1:**
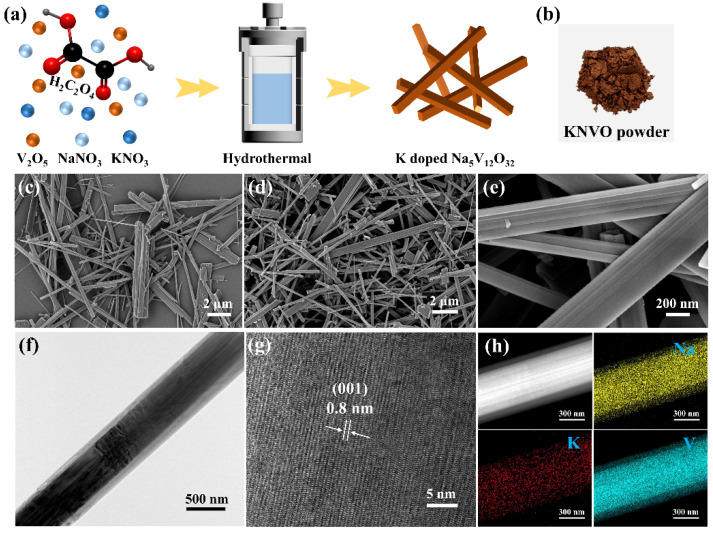
(**a**) Schematic illustration of the production of KNVO. (**b**) Photograph of KNVO powder. (**c**) SEM image of NVO. (**d**,**e**) SEM images of KNVO. (**f**,**g**) TEM and HRTEM images of KNVO. (**h**) STEM and elemental mapping images of KNVO.

The preparation of the KNVO is illustrated in Figure 1a, where V_2_O_5_, NaNO_3_, KNO_3_, and H_2_C_2_O_4_ were used as the vanadium source, sodium source, dopant, and reducing agent, respectively. After a simple hydrothermal process, the orange product with a mixed valence state of V^5+^ and V^4+^ was obtained (Figure 1b). The morphology and nanostructure were first investigated using SEM and TEM. As displayed in Figure 1b and Figure S1, the NVO shows a smooth nanobelt structure with a length of several micrometers. After the addition of the dopant K, the nanobelt shape of the KNVO is maintained, demonstrating that the crystalline structure of the NVO has not changed in the presence of K (Figure 1c,d and Figure S2). The TEM image (Figure 1f) confirms that the as-prepared KNVO nanobelt structure is a monoclinic single crystal with high crystallinity. As shown in Figure 1g, a lattice fringe with d-spacing of 0.8 nm can be distinguished from the high-resolution TEM (HRTEM) image, which is well matched to the (001) plane of Na_5_V_12_O_32_ [27,30]. The high-angle, annular dark-field scanning TEM (HAADF-STEM) and the related energy-dispersive X-ray spectroscopy (EDX) elemental mapping images are shown in Figure 1h, confirming that the Na, K, and V elements are homogeneously dispersed throughout the entire layered structure. The EDX result demonstrates that the K element was successfully doped into the KNVO nanobelts. The unique one-dimensional structure can improve the specific surface area, reduce the Zn^2+^ ions–electrons diffusion distance, and alleviate the lattice expansion–contraction stress upon the Zn^2+^ intercalaction and deintercalation process, thus enhancing the rate capability and cyclability.

**Figure 2:**
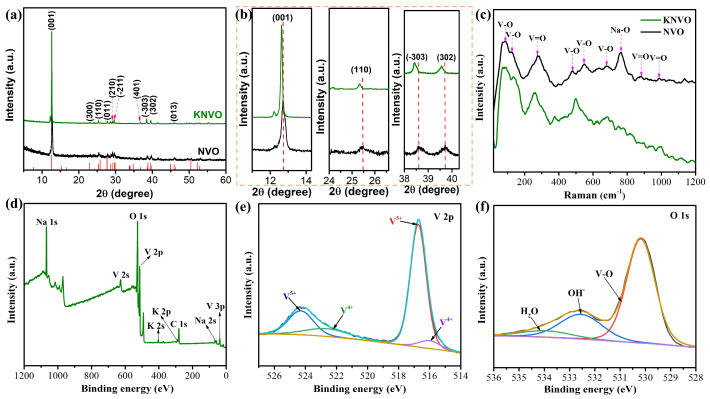
(**a**) XRD patterns of KNVO and NVO and (**b**) their high-resolution patterns of (001), (110), (−303), and (302) planes. (**c**) Raman spectra of KNVO and NVO. (**d**) XPS spectrum of KNVO. XPS spectra of (**e**) V 2p and (**f**) O 1s, respectively.

The phase state and purity of the as-synthesized KNVO, KNVO-2.5, KNVO-7.5, and NVO samples were characterized by XRD patterns, as displayed in Figure 2a,b and Figure S3. It is noted that all diffraction peaks are well matched with the standard XRD data card of Na_5_V_12_O_32_ (JCPDS card no. 24-1156), demonstrating the high purity and crystal of the electrode materials [27,30]. The peaks located at 12.8, 23.2, 25.6, 27.7, 29.1, 29.7, 36.9, 38.6, 39.7, and 46.2° are assigned to the (001), (300), (110), (011), (210), (−211), (401), (−303), (302), and (013) lattice planes of the NVO. Obviously, the (001), (110), (−303), and (302) planes of the synthesized KNVO, KNVO-2.5, and KNVO-7.5 have a slight shift to the low angle region compared to the NVO, showing that the K has successfully doped into the NVO and further enlarged the interlayer spacing. The increased interlayer spacing could promote the Zn^2+^ ions migration and alleviate the volume variation during the Zn^2+^ intercalation and deintercalation process, thus boosting the rate capability and cyclability. The Raman spectra of the KNVO and NVO are displayed in Figure 2c. The peaks characterized at 90, 129, 278, 481, 550, 680, 884, and 988 cm^−1^ are assigned to the stretching vibration mode of [VO_n_] bonds in the NVO [29,37,39]. Specifically, the low frequency peaks at 90 and 129 cm^−1^ are associated with the bending vibration modes of V–O bonds [13,29]. The peaks at 278, 884, and 988 cm^−1^ are ascribed to the bending vibration modes of V–O bonds [31,33]. The peaks sited at 481, 550, and 680 cm^−1^ are corresponded to the stretching vibration modes of V–O bonds [24,34]. Meanwhile, the signals at 764 cm^−1^ can be associated with Na–O bonds (the corner-sharing oxygen among the VO_6_, VO_5_ polyhedral and Na^+^ ions) [18,32]. The Raman spectrum of the KNVO displays similar peaks with some shift, indicating the K doped into the interlayer structure in accordance with the XRD results. In addition, the surface chemical composition of the KNVO was further examined by the X-ray photoelectron spectroscopy (XPS) (Figure 2d–f). As depicted in Figure 2d, in addition to the distinctive peaks of Na, V, and O, additional peaks belonging to K are also present in the survey spectrum of the KNVO, confirming the successful doping of K. As presented in Figure 2e, the V 2p spectra demonstrates two separated peaks of V^5+^ (524.3 and 516.7 eV) and V^4+^ (522.6 and 515.8 eV). The fitted area ratio of V^4+^ to V^5+^ is about 18.6%, which is higher than that the expected value of 9.1% in the NVO, demonstrating that partial V^5+^ was transformed to V^4+^ due to the doping of K, which could be favorable for improving the electron migration kinetics [17,18,40]. In addition, the high-resolution O 1s spectra (Figure 2f) can be distinguished into three peaks sited at 530.1, 532.3 eV, and 533.9 eV. These peaks are distinguished from the O adsorbed on the sample surface (531.5 eV), so it should be ascribed to the V–O bonds, OH^−^, and H_2_O [14,18,41,42], respectively.

**Figure 3:**
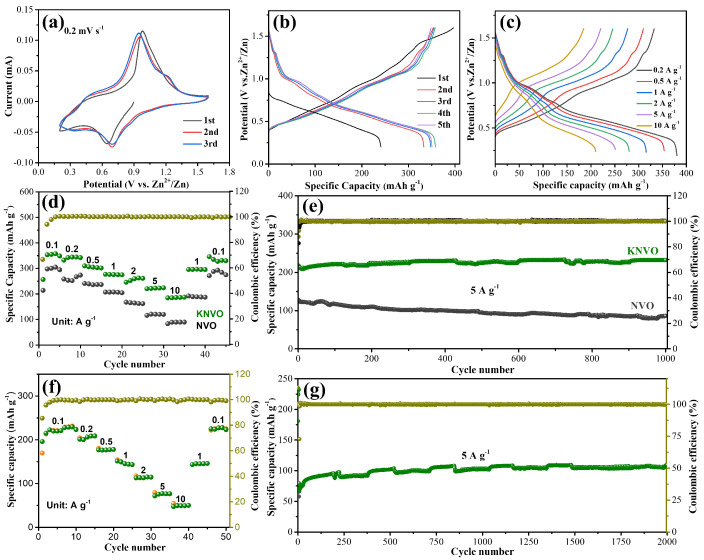
(**a**) CV curves of KNVO at 0.2 mV s^−1^. (**b**) The first five GDC profiles of KNVO at 0.1 A g^−1^. (**c**) The GDC curves of KNVO from 0.2 to 10 A g^−1^. (**d**) Rate capability of KNVO and NVO cathodes. (**e**) Long-term cycling stability of KNVO and NVO cathodes. (**f**) Rate capability of KNVO at high mass loading. (**g**) Cyclic life of KNVO at high mass loading.

To further examine the electrochemical performance of KNVO and NVO, CR2032 coin-type cells were fabricated using 3 M Zn(CF_3_SO_3_)_2_ as the electrolyte. The CV profiles of the KNVO and NVO were first tested at 0.2 mV s^−1^ with a voltage span of 0.2–1.6 V, as displayed in Figure 3a and Figure S4a. Obviously, both of the KNVO and NVO samples display similar CV profiles, with two pairs of oxidation reduction peaks sited at 0.96–0.69 V and 0.46–0.32 V, respectively, indicating that a multistep redox reaction happened during the Zn^2+^ storage and release process. Obviously, the initial CV curve is significantly different from the next two curves, demonstrating that an irreversible reaction happened during the initial discharging and charging procedure. The first five galvanostatic discharge–charge (GDC) profiles of the KNVO and NVO are shown in Figure 3b and Figure S4b. The GDC curves display two pairs of platforms at 0.99–0.58 V and 0.67–0.92 V, respectively, in agreement with the CV curves. The GDC curves and rate capabilities of the KNVO, KVNO-2.5, KVNO-7.5, and NVO samples at various currents are demonstrated in Figure 3c,d, Figure S5 and Figure S6, respectively. The reversible average capacities of the KNVO are 353.5, 344.4, 306.5, 275.8, 259.4, 222.8, and 185.8 mAh g^−1^ at currents of 0.1, 0.2, 0.5, 1, 2, 5, and 10 A g^−1^, respectively, which are superior to those of the NVO (300.5, 253.3, 239.4, 207.1, 165.6, 120.4, and 89.4 mAh g^−1^), KNVO-2.5 (336.1, 306.2, 252.7, 225.3, 194.5, 152.5, and 130.1 mAh g^−1^), and KNVO-7.5 (341.8, 314.2, 266.3, 237.5, 208.3, 170.7, and 145.5 mAh g^−1^). In addition, as the current swithes back to 0.1 A g^−1^, a high capacity of 346 mAh g^−1^ can still be maintained, showing high reversibility. Figure 3e presents the long-term cyclability of the KNVO and NVO at 5 A g^−1^. The KNVO electrode presents an initial capacity of 217.5 mAh g^−1^, which is larger than that of the NVO. After 1000 cycles, the capacity of the KNVO cathode retains a stable capacity of 231.8 mAh g^−1^, demonstrating superior cycle stability. However, for the NVO electrode, the initial capacity is 126.9 mAh g^−1^, which quickly decreases to 86.1 mAh g^−1^. These results demonstrate that the KNVO possesses both remarkable rate performance and cyclability, which should be ascribed to the K doping into the interlayer spacing and acting as pillar ions, promising a fast Zn^2+^ transport pathway and improving the structural stability. Additionally, the KNVO cathode also displays excellent electrochemical properties at high mass loading. As shown in Figure 3f, even under a mass loading of 5.3 mg cm^−2^, a high reversible capacity of 220.5 mAh g^−1^ (equal to 1.2 mAh cm^−2^) can still be delivered. In addition, a reversible capacity of 76.7 mAh g^−1^ can still be maintained when the current is raised to 5 A g^−1^. As presented in Figure 3g, the high mass loading electrode also displays an excellent cycling stability with no apparent capacity degradation after 2000 cycles at 5 A g^−1^, demonstrating the practical application of the KNVO.

**Figure 4:**
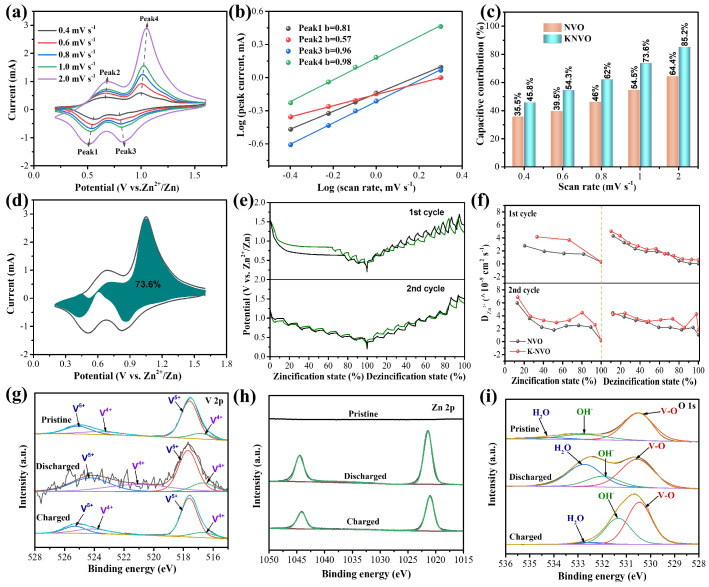
(**a**) CV profiles of KNVO electrode. (**b**) Log (*v*) versus log (*i*) plots at certain peak currents. (**c**) The capacitive behavior contribution ratios of KNVO and NVO electrodes. (**d**) Capacitive fraction of KNVO cathode calculated at 1 mV s^−1^. (**e**) GITT profiles of KNVO and NVO electrode at the 1st and 2nd GDC curves. (**f**) Zn^2+^ diffusion coefficients of KNVO and NVO at the 1st and 2nd GDC curves. Ex situ XPS test of (**g**) V 2p, (**h**) Zn 2p, and (**i**) O 1s spectra of KNVO electrode.

To further investigate the electrochemical evolution of KNVO, the CV curves were tested with various sweep rates. As demonstrated in Figure 4a, two distinct couples of oxidation reduction peaks are found in all CV curves from 0.4 to 2.0 mV s^−1^, suggesting that the electrode occurs a multi-step insertion–extraction process. The shape of CV profiles remains similar when increasing the scan rate, except that redox peaks shift in a certain direction due to the polarization effect. Generally, the power–law correlation between peak current (*i*) and sweep rate (*v*) is obeyed to the equation of $i=av^{b}$ (*a* and *b* represent the adjustment coefficients), where the *b* value is in the range of 0.5 to 1 [5,14,43]. When the *b* value is close to 0.5 and 1, it indicates that the redox reaction process is dominated by a faradaic diffusion behavior and capacitive behavior, respectively [5,17,44]. By fitting plots of log(*i*) and log(*v*), these peaks 1, 2, 3, and 4 have *b* values of 0.81, 0.57, 0.96, and 0.98, respectively (Figure 4b), which is superior to that of the NVO cathode (Figure S7). Additionally, the contribution ratios of capacitive behavior ($k_{1}v$) and diffusion dominated behavior ($k_{2}v^{1/2}$) can be calculated from the formula $i=k_{1}v+k_{2}v^{1/2}$ [37,44]. As presented in Figure 4c,d, the capacitive contribution of the KNVO cathode ranges from 45% to 85.2% at a sweep rate of 0.4 to 2 mV s^−1^, which is higher than that of the NVO cathode (Figure S8, from 35.5% to 64.4%). These results show that the KNVO cathode is mainly dominated by a capacitive behavior during the Zn^2+^ insertion–extraction process, which could be ascribed to the pre-intercalation of the K, reducing the Zn^2+^ migration barrier and allowing for the fast Zn^2+^ insertion–extraction. To confirm this, the Zn^2+^ diffusion coefficient of the KNVO and NVO electrodes during the 1st and 2nd GDC processes were calculated from the galvanostatic intermittent titration technique (GITT) curves, the results of which are displayed in Figure 4e,f. Obviously, the Zn^2+^ diffusion coefficient can be calculated to be 1.2 × 10^−9^ to 7.2 × 10^−9^ cm^−2^ s^−1^ in the 1st cycle and 2nd cycle, and the Zn^2+^ diffusion coefficient of KNVO cathode is superior to that of the NVO cathode, which manifests the outstanding reaction kinetics in the KNVO. These results are in agreement with the EIS test (Figure S9) and declare its superb rate performance.

To further examine the redox reaction kinetics of KNVO in AZIBs, the valence changes upon the first discharge–charge cycle were characterized by the ex situ XPS experiments (Figure 4g–i and Figure S10). As shown in Figure 4g, the V^4+^ signal is enhanced after discharging to 0.3 V compared to the pristine state, demonstrating the reduction of V^5+^ by the intercalation of Zn^2+^. When charged to 1.6 V, the intensity of the V^4+^ signal is decreased again, manifesting that the V^4+^ is reversibly oxidized [2,13,17]. This phenomenon is in accordance with the XPS result of Zn 2p. As presented in Figure 4h, no Zn 2p signal could be identified in the pristine state, whereas two distinct peaks sited at 1021.5 and 1044.5 eV are observed in the discharged state, manifesting the successful insertion of Zn^2+^ [18,19,34]. Upon charging to 1.6 V, the Zn^2+^ signal becomes weaker, demonstrating the extraction of Zn^2+^. The weak signal of Zn^2+^ should be caused by remaining electrolyte adsorption. As depicted in Figure 4i, in the spectrum of O 1s, the signal of H_2_O molecules is enhanced upon discharge to 0.3 V, suggesting the coinsertion of the Zn^2+^ and H_2_O molecules [19,24,45]. In the charging process, the peak becomes weaker as the Zn^2+^ deintercalated. The XPS results prove the reversible coinsertion and extraction of the Zn^2+^ and H_2_O molecules upon the redox reaction. The XPS spectrum of KNVO after 100 cycles was also determined to detect the valence state evolution (Figure S11). As shown in Figure S11b, the peak area ratio of V^4+^ to V^5+^ is increased to 39.9% after 100 cycles, which could be ascribed to the insertion of Zn^2+^. This result is consistent with the ex situ XPS result (Figure S4g–i), further demonstrating the highly reversible redox reaction.

**Figure 5:**
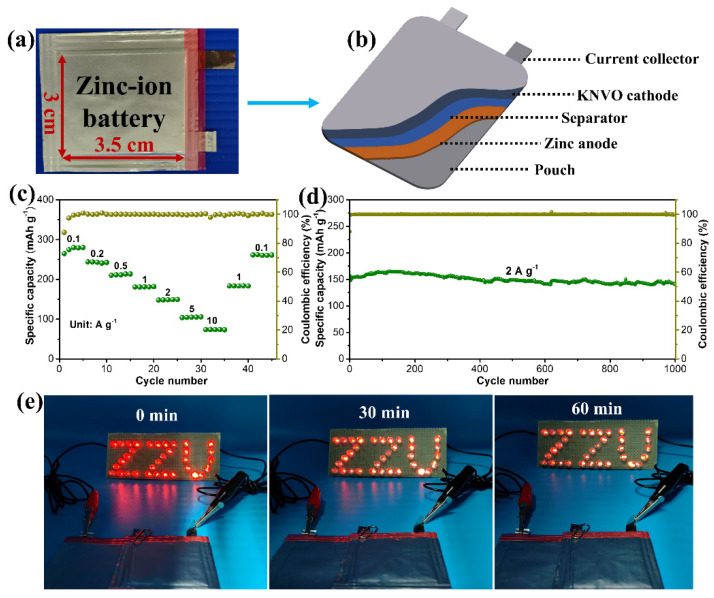
(**a**) Photograph of a 3 × 3.5 cm^2^ pouch cell. (**b**) Illustration of the structure of AZIB pouch cell. (**c**) Rate capability of Zn//KNVO pouch cell. (**d**) Cyclic life of Zn//KNVO pouch cell. (**e**) Photo of an LED panel driven by two series-connected Zn//KNVO cells.

To demonstrate the practical application of KNVO, a 3 × 3.5 cm^2^ pouch cell was fabricated (Figure 5a). As illustrated in Figure 5b, the pouch cell employed a KNVO-coated, stainless steel film as a cathode and a pure zinc plate as an anode, with a whatman GF-D glass filter between them as a separator. As displayed in Figure 5c, the constructed pouch cell also offers a superior rate capability, which can exhibit a reversibele capacity of 275.6 mAh g^−1^ and 73.9 mAh g^−1^ at 0.1 and 10 A g^−1^, respectively. Notably, the Zn//KNVO pouch cell also demonstrated a remarkable cyclability with a 89.7% capacity retention and a stable coulombic efficiency (>90%) after 1000 cycles at 2 A g^−1^ (Figure 5d). To intuitively demonstrate the practicality of the Zn//KNVO pouch cell, two charged cells connected in a series can power a LED panel for several hours (Figure 5e). Based on the whole weight of the pouch cell (including the weight of two electrodes, electrolyte, separator, and packages), the energy density of the pouch cell was estimated to 96.3 Wh kg^−1^ [46]. Considering the cost of the cathode, separator, current collector, electrolyte, Zn anode, and Al package, the cost of the Zn//KNVO pouch cell is about USD 93.8 kWh^−1^ [46,47]. Although the cost is higher than the previously reported Ni–Zn battery, it is still has price advantage over the lead–acid battery (USD 150–500 kWh^−1^) and Ni–MH battery (USD 200–729 kWh^−1^) [46,47,48]. If a more reasonable N/P ratio is optimized, the cost can be further reduced.

## 4. Conclusions

In conclusion, a Na_5_V_12_O_32_ nanobelt with K pre-intercalated material was successfully prepared by a facile hydrothermal process. When employed as a cathode for AZIBs, the produced material exhibits a competitive electrochemical performance with a reversible specific capacity of 353.5 and 185.8 mAh g^−1^ at 0.1 and 10 A g^−1^, respectively. The cathode can also demonstrate an impressive capacity of 231.8 mAh g^−1^ after 1000 cycles at a current of 5 A g^−1^. More importantly, even under a mass loading of 5.3 mg cm^−2^, the KNVO cathode can still provide satisfactory capacity and remarkable cyclability. The superior Zn^2+^ storage capability is ascribed to the doping of K, which can reduce the Zn^2+^ diffusion barrier, enhance the electroconductibility, and inhibit the structural collapse. In addition, the elaborate construction of the Zn//KNVO pouch cell can deliver an energy density of 96.3 Wh kg^−1^ with the low cost of USD 93.8 kWh^−1^. This work presents a promising method for creating highly durable and cost-effective AZIBs for large-scale energy storage applications.

## Data Availability

The original contributions presented in the study are included in the article/Supplementary Material, further inquiries can be directed to the corresponding author.

## Supplementary Materials

The following supporting information can be downloaded at: https://www.mdpi.com/article/10.3390/ma17194703/s1, Figure S1: SEM images of NVO cathode; Figure S2: SEM image of KNVO cathode; Figure S3: (a) XRD patterns of KNVO-2.5, KNVO-7.5 and NVO and (b) their high-resolution pattern of (001), (110), (−303) and (302) planes; Figure S4: (a) The first three CV curves of NVO. (b) The first five GDC curves of NVO at 0.1 A g^−1^; Figure S5: The GDC profiles of NVO at different current densities; Figure S6: Rate capability of KNVO-2.5, KVNO-7.5 and NVO; Figure S7: (a) CV profiles of NVO at different current densities. (b) Log (*v*) versus log (*i*) plots at specific peak currents; Figure S8: Capacitive fraction of KNVO cathode calculated at a scan rate of 1 mV s^−1^; Figure S9: EIS Nyquist plots of KNVO and NVO; Figure S10: Ex situ XPS spectra of survey in the pristine, discharged, and charged states; Figure S11: (a) XPS spectra of KNVO in the pristine and after 100 cycles. (b) XPS spectrum of V 2p after 100 cycles.
