# Supplementary material for: Boosting Zn2+ Storage Kinetics by K-Doping of Sodium Vanadate for Zinc-Ion Batteries
Source: Materials (Basel). 2024 Sep 25;17(19):4703. doi: 10.3390/ma17194703 (PMC11478306; doi:10.3390/ma17194703)
Supplement: Supplementary file 1 [file materials-17-04703-s001.zip › materials-3191203-supplementary.pdf]

Supplementary Materials

Boosting Zn^{2+} Storage Kinetics by K-Doping of Sodium Vanadate for Zinc-Ion Batteries

Mengting Jia, Chen Jin, Jiamin Yu and Shaohui Li *

School of Materials Science and Engineering, Zhengzhou University, Zhengzhou 450001, China
* Correspondence: shaohuili@zzu.edu.cn

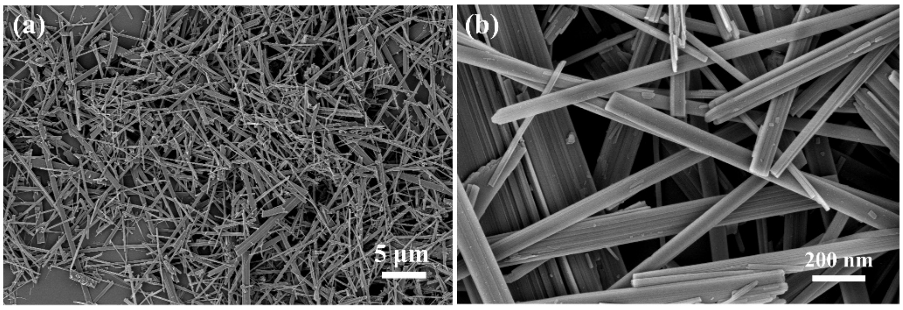

Figure S1. SEM images of NVO cathode.

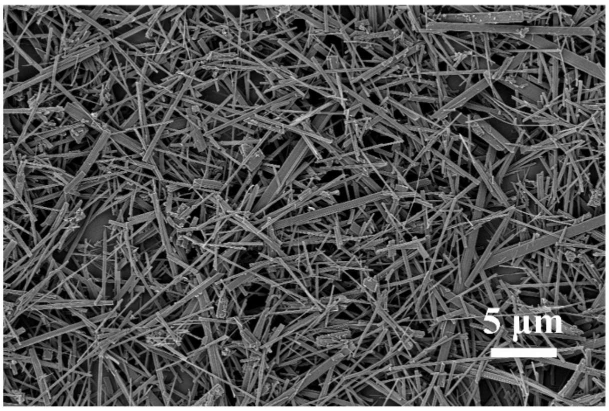

Figure S2. SEM image of KNVO cathode.

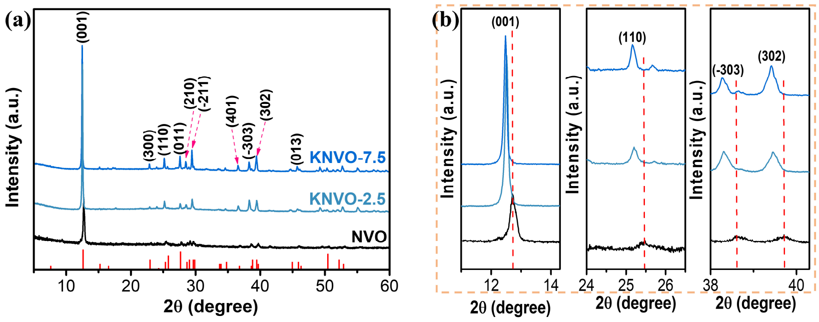

Figure S3. (a) XRD patterns of KNVO-2.5, KNVO-7.5 and NVO and (b) their high-resolution patterns of (001), (110), (-303) and (302) planes.

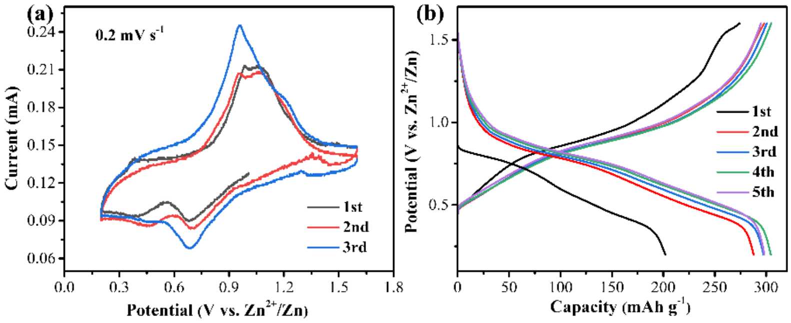

Figure S4. (a) The first three CV curves of NVO. (b) The first five GDC curves of NVO at 0.1 A g^{-1}.

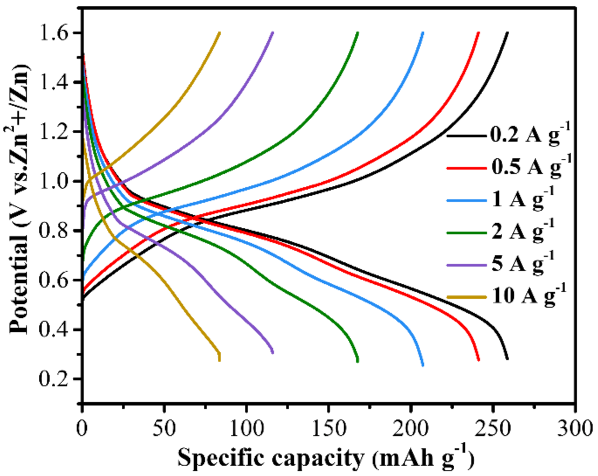

Figure S5. The GDC profiles of NVO at different current densities.

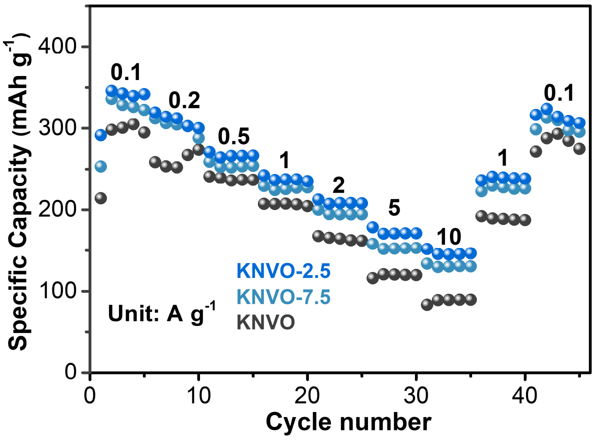

Figure S6. Rate capability of KNVO-2.5, KNVO-7.5 and NVO electrodes.

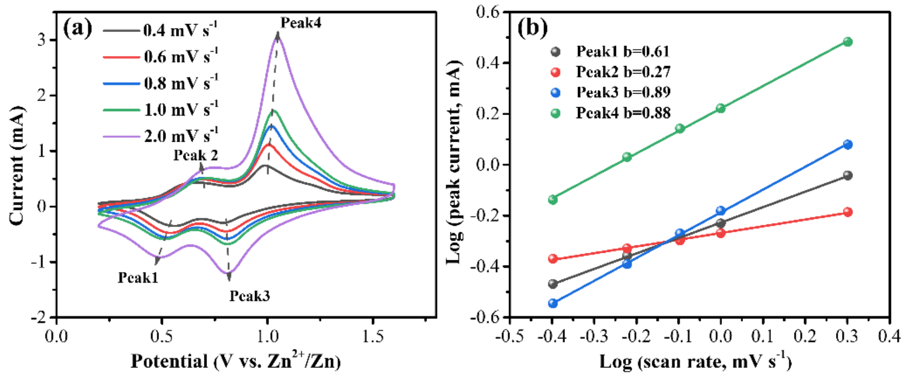

Figure S7. (a) CV profiles of NVO at different current densities. (b) Log (*v*) versus log (*i*) plots at specific peak currents.

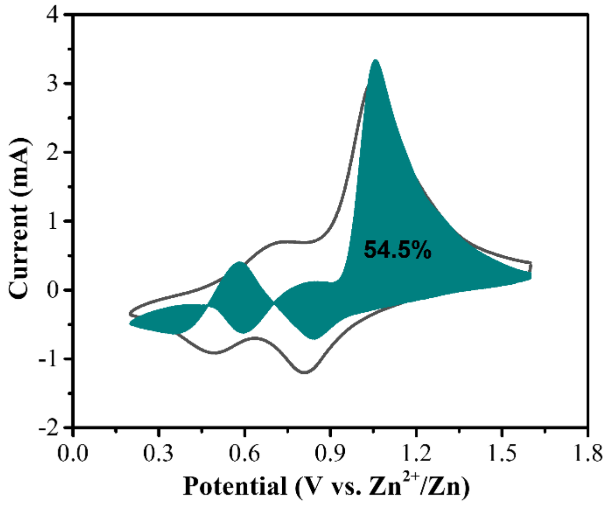

Figure S8. Capacitive fraction of KNVO cathode calculated at a scan rate of 1 mV s^{-1}.

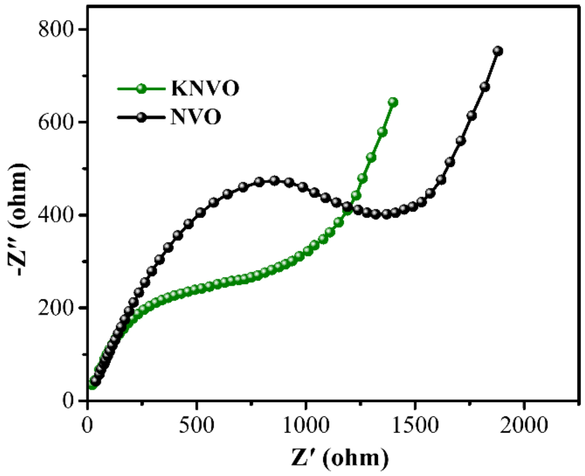

Figure S9. EIS Nyquist plots of KNVO and NVO.

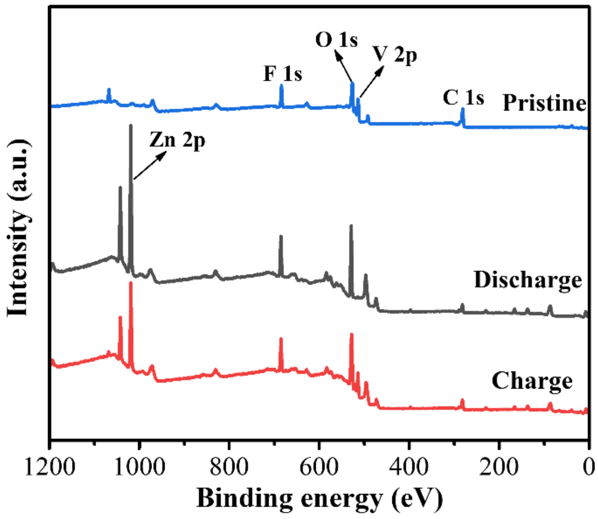

Figure S10. Ex-situ XPS spectra of survey in the pristine, discharged, and charged states.

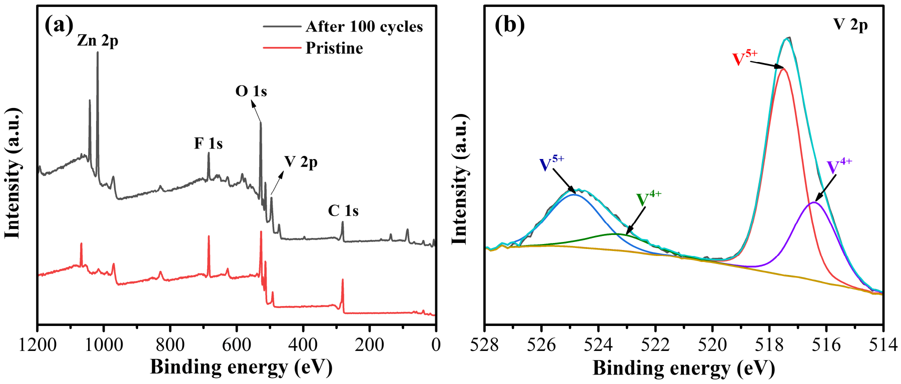

Figure S11. (a) XPS spectra of KNVO in the pristine and after 100 cycles. (b) XPS spectrum of V 2p after 100 cycles.
